# Opposite associations of collective narcissism and in-group satisfaction with intergroup aggression via belief in the hedonistic function of revenge

**DOI:** 10.1371/journal.pone.0247814

**Published:** 2021-03-10

**Authors:** Karolina Dyduch-Hazar, Blazej Mrozinski

**Affiliations:** Institute of Psychology, SWPS University of Social Sciences and Humanities, Warsaw, Poland; Univeristy of Padova, ITALY

## Abstract

We investigated whether collective narcissism (i.e., believing that the in-group is exceptional but insufficiently recognized by others) and in-group satisfaction (i.e., believing that the in-group is a source of satisfaction) have opposite, unique associations with intergroup aggression via belief in the hedonistic function of revenge (i.e., an expectation of emotional reward from harming others in response to feeling oneself harmed). Results of two studies conducted in Poland (*N* = 675) found that collective narcissism is positively related to belief in the hedonistic function of revenge, whereas in-group satisfaction is negatively related, and both are related to intergroup aggression. These relationships were found only when the overlap between collective narcissism and in-group satisfaction was partialled out. The results shed a new light on the mechanisms linking in-group positivity to out-group derogation, and highlight the importance of investigating revenge motivations in the intergroup relations.

## Introduction

Although revenge is a common human motivation [1], it has been relatively understudied in the domain of intergroup relations. The present research we investigated intergroup consequences of belief that “revenge is sweet” by linking it to the beliefs about social identity and intergroup aggression. More specifically, we examined whether collective narcissism, i.e., a belief that the exceptionality of one’s own group is insufficiently appreciated by others [2] and in-group satisfaction, i.e., a belief that the in-group is the reason to be proud [3] make distinct predictions for intergroup aggression via belief in the hedonistic function of revenge, i.e., an expectation of a momentary good feeling from harming others in response to feeling oneself harmed: Collective narcissism is positively associated, whereas in-group satisfaction is negatively associated, with belief in the hedonistic function of revenge and, therefore, with intergroup aggression (Hypothesis 1). Because previous findings showed that collective narcissism and in-group satisfaction act as mutual suppressors [2], we also tested whether collective narcissism and in-group satisfaction mutually suppress each other’s associations with belief in the hedonistic function of revenge (Hypothesis 2).

We propose that belief in the hedonistic function of revenge might be related to collective narcissism and in-group satisfaction because they explain, unlike other similar constructs (patriotism vs. nationalism, [4]; constructive vs. blind patriotism, [5]; in-group attachment vs. in-group glorification, [6]), the mechanisms linking in-group positivity to out-group derogation [2] and are related to different self-evaluations [7]. Revengeful motivation as a drive for aggressive behaviour against out-groups has not yet been studied with relation to collective narcissism and in-group satisfaction. However, such a belief might explain perseverance of aggressive tendencies among people who are narcissistic about the in-group, and lack of such tendencies amongst satisfied in-group members.

### Collective narcissism and in-group satisfaction are associated with different emotional profiles and attitudes towards out-groups

We interpret collective narcissism and in-group satisfaction as alternative beliefs people may hold about the value of the social identity they share ([2]; see [8] for an alternative interpretation). Collective narcissism focuses on the resentment for unrecognized greatness of the in-group, whereas in-group satisfaction emphasizes the concern about the in-group welfare. They overlap, but result in different attitudes towards out-groups when their common variance is partialled out [9, 10]).

Collective narcissism in its residual form is a group-based entitlement without the sense of belonging to a valuable in-group. In-group satisfaction in its residual form is a positive evaluation of the in-group, independent of external recognition and resilient to threats. As such, residual collective narcissism is related to out-group derogation, whereas residual in-group satisfaction is linked to out-group tolerance [9, 11, 12]. Those two beliefs about the in-group are also associated with different self-evaluations and emotional profiles [7].

Collective narcissism is associated with low self-esteem [10], low personal control [13] and a lack of social connectedness [7]. Collective narcissists are predisposed to experience aversive affective states such as anger or anxiety [7]. Negative affect is likely to motivate them to seek out aggression’s mood improving qualities [14], thus explaining their aggressive tendencies [2, 15]. In contrast to collective narcissism, in-group satisfaction is positively related to self-esteem [10], pro-sociality, and psychological well-being [7]. Moreover, positive social identification, akin to in-group satisfaction, has also been linked to better mental health [16, 17]. Lastly, greater in-group satisfaction is associated with stronger beliefs that the positive characteristics of individuals should be used to enhance the value of the in-group [18]. Collective narcissism and in-group satisfaction often act as mutual suppressors of their opposite associations with variables pertaining to intergroup relations: attitudes toward out-groups [19], willingness to accept past transgressions of the in-group [20]; solidarity with in-group members in time of crisis [21]; as well variables related to individual characteristics such as self-esteem [10]. Taking all these findings together, we expect collective narcissism and in-group satisfaction to mutually suppress each other’s associations with belief in the hedonistic function of revenge.

### Belief in the hedonistic function of revenge, narcissism and aggression

Although results are mixed with respect to whether revenge is in fact a pleasant experience [22–24], people expect to “reap hedonistic rewards” from revenge [24]. This expectation is expressed by a popular saying “revenge is sweet,” which we operationalize as the belief in the hedonistic function of revenge: an expectation of momentary good feeling from harming others in response to feeling oneself harmed. Such a belief is correlated with joy at the misfortunes of others, low agreeableness, and high neuroticism [25]–dispositions showed to be associated with narcissism. Finally, such a belief motivates aggression. There are reasons to expect that narcissism, the individual desire for external validation of one’s inflated self-view [26], may be related to aggression via belief in the hedonistic function of revenge. Narcissists tend to be envious [27] and hateful [28], and people who possess such traits are more inclined to have revengeful motivations [29]. Narcissism is also associated with vindictiveness [30–32] and retaliatory behaviour [33], as well as disconnection between self and reward [34]. Narcissists may be therefore especially inclined to engage in actions that may be potentially rewarding.

Since the concept of collective narcissism extends into the intergroup domain the concept of individual narcissism, many correlates of collective narcissism parallel the correlates of individual narcissism in an intergroup context [2]. As such, collective narcissism reliably predicts rejoicing in suffering of out-groups [35] and intergroup hostility [10, 12, 36]. Specifically, it is associated with persistent hypersensitivity to threats to the in-group’s privileged position [36], chronic resentment for insufficient recognition of the in-group [2], and negative emotionality [7].

Collective narcissists may be particularly inclined to believe that revenge gives good feeling, and especially attracted to experiences that may temporarily improve their mood, such as aggression [14]. Holding such a belief may be a way of coping with tension and a way of justifying aggression against out-groups, especially when the privileged position of the in-group is threatened or past harm to the in-group is made salient.

In-group satisfaction, on the other hand, is linked to intergroup tolerance [9, 10, 36] and psychological well-being [7]. Satisfied in-group members who are resilient to intergroup threats [11], and whose emotionality is rather positive [7], may not be inclined to believe in the hedonistic function of revenge.

### Overview

We tested two hypotheses: Collective narcissism and in-group satisfaction have opposite indirect associations with intergroup aggression via belief in the hedonistic function of revenge (Hypothesis 1) and mutually suppress each other’s relationships with belief in the hedonistic function of revenge (Hypothesis 2). Suppression occurs when one variable increases the predictive validity of another variable and when a direct and indirect (via suppressor) link between two variables have opposite signs—a significant positive predictor becomes a significant negative predictor and vice versa [37]. Since, we tested the mutual suppression, we expected collective narcissism and in-group satisfaction to mutually enhance their predictive validities and change signs [38].

Two studies were conducted in Poland. Study 1 concentrated on aggressive behavioural intentions against British people. Although the majority of Poles hold British people in high regard [39], relations between two nations include resentment [40]. In order to examine whether collective narcissism predicts belief in the hedonistic function of revenge especially when past harm to the in-group is made salient, we manipulated intergroup resentment by reminding participants of the broke of Polish-British military alliance. Study 2 focused on symbolic aggression against Syrian refugees since the majority of Poles do not have a positive opinion of Syrian refugees [41]. Syrian refugees are perceived as a threat to social cohesion and economic resources [42] and rejected [43].

## Study 1

### Method

#### Participants

We used the power Mediation package [44] for R to calculate the minimum required sample size based on the Sobel test. Regression coefficients were set to .15 for the path between the predictor and the mediator, as for the path between the mediator and the outcome. Standard deviations of the predictors and mediators were set to .50. The smallest estimated sample size to discover the indirect effect was *N* = 398 (power = .80, alpha = .05).

401 Polish nationals (257 female, 144 male; Age: *M* = 38.09; *SD* = 12.19, range: 18–75), participated in a study conducted by Ariadna Research Panel (https://panelariadna.pl). All research procedures were approved by the Research Ethics Committee at SWPS University of Social Sciences and Humanities. The consent was obtained in written form (17/2019).

#### Procedure

After providing their informed consent, participants took part in a study ostensibly examining associations between personality traits, cognitive load, and attitudes towards out-groups. First, participants responded to the scales assessing collective narcissism and in-group satisfaction. Then, they were randomly assigned to one of two conditions: Being reminded about past intergroup resentment (*N* = 182) vs. control (*N* = 219). Participants were presented with a text on Polish-British historical relations and asked to highlight and count the letters that were specific to Polish language in less than a minute. They were told that the task was a measure of their ability to concentrate. The texts in both conditions were identical with one exception: The text in the experimental condition included information on the alliance pact the British broke with Poland in 1939. Next, participants answered the manipulation check items, and they completed the Hedonistic Belief About Revenge Scale and a measure of behavioural inclinations toward British. Finally, they were thanked and debriefed.

### Materials

The response scale to all measures ranged from 1 (*totally disagree*) to 6 (*totally agree*).

**Manipulation check** was measured by asking participants to what extent British people: “harmed”, “humiliated”, “supported” (reversed), and “helped” (reversed) Polish people, *α* = .86, *M* = 3.21, *SD* = 1.10.

**Collective narcissism** was measured by the 5-item Collective Narcissism Scale ([45]; e.g. “Polish people deserve special treatment”), *α* = .88, *M* = 3.48, *SD* = 1.07.

**In-group satisfaction** was measured by the 4-item In-group satisfaction subscale of the In-group Identity Scale ([3, 46]; e.g. “I’m proud to be Polish”), *α* = .94, *M* = 4.59, *SD* = 1.03.

**Belief in the hedonistic function of revenge** was measured by the 5-item Hedonistic Belief About Revenge Scale (e.g. “Revenge gives me pleasure”), *α* = .97; *M* = 1.98, *SD* = 0.97.

**Intergroup aggression** was measured by 4 items (based on [46]). Participants were asked to indicate to what extent they would like to engage in each of the following behaviours towards British people: “harm”, “humiliate”, “offend”, “attack”; *α* = .88; *M* = 1.34; *SD* = 0.62.

### Results

Zero-order correlations between variables are summarized in Table 1. Yet participants in the experimental condition were more prone to believe that Polish-British relations include resentment (*M* = 3.69, *SD* = .56) than participants in the control condition (*M* = 3.08; *SD* = .43) *t*(399) = 12.44, *p* < .001, the experimental manipulation did not moderate any associations in the model. Although participants in the experimental manipulation tended to be more aggressive against British (*b* = .14, *SE* = .06, *p* = .02), the experimental manipulation did not affect mean score for the belief in the hedonistic function of revenge, *p* = .342. Analyses with research condition entered as the moderator of the associations in the model are summarized in S1 Table.

**Table 1:** Zero-order correlations among variables in Studies 1 and 2.

	Study 1			Study 2		
Variables	1.	2.	3.	1.	2.	3.
**1. Collective narcissism**	---					
**2. In-group satisfaction**	.64***	---		.54***		
**3. Belief in the hedonistic function of revenge**	.04	-.21***	---	.11	-. 09	
**4. Intergroup aggression**	.15**	-.07	.30***	. 21*	. 11	. 29***

* *p* < .05,

** *p* < .01,

***p < .001.

In order to test the Hypothesis 1 that collective narcissism and in-group satisfaction have opposite indirect associations with intergroup aggression via belief in the hedonistic function of revenge, we ran a path analysis in which collective narcissism and in-group satisfaction were entered as correlated predictors, belief in the hedonistic function of revenge as mediator, and intergroup aggression as outcome.

Analyses were performed in MPlus 8.4 with maximum likelihood estimation. We analysed the hypothesized relationships using a mediation model with two concurrent predictors to partial out variance shared by collective narcissism and in-group satisfaction. When the covariation between in-group satisfaction and collective narcissism was controlled, the path between collective narcissism and belief in the hedonistic function of revenge became positive and significant.

**Fig 1:**
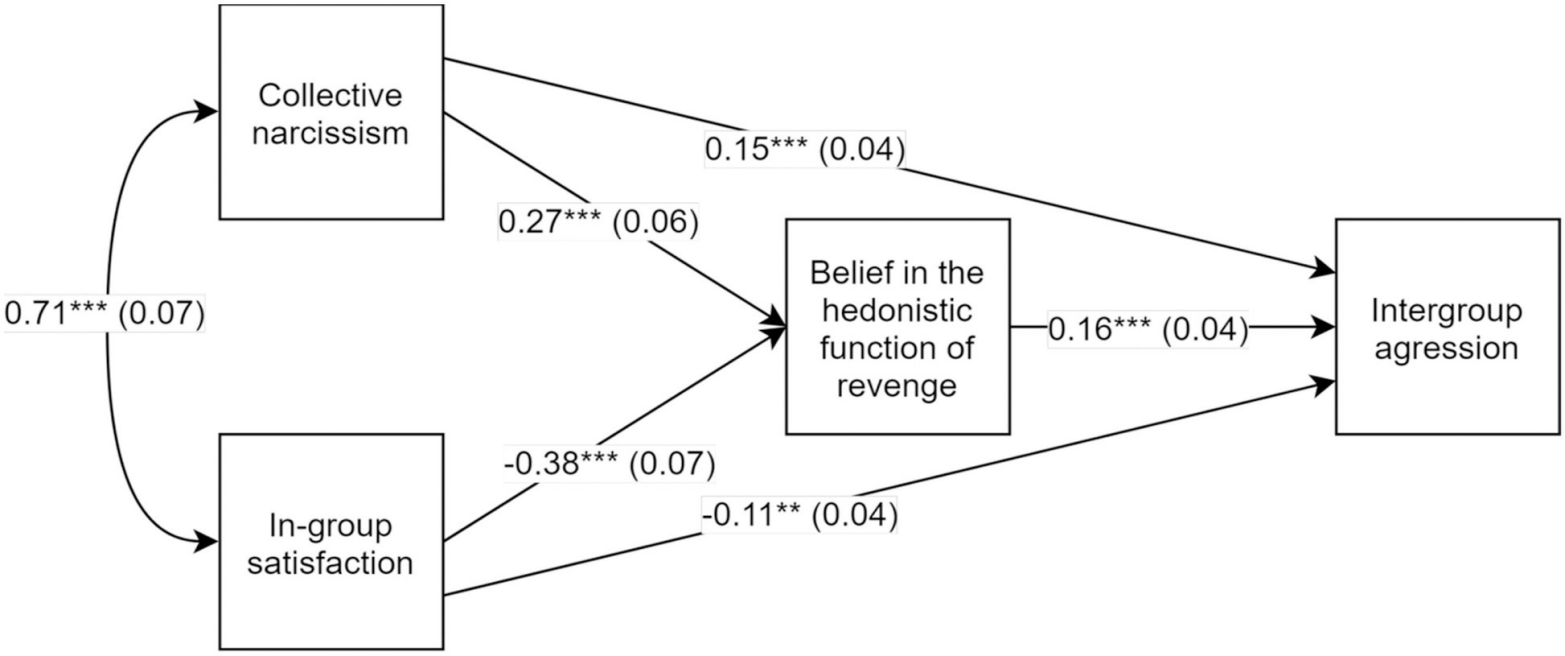
Unstandardized path coefficients, Study 1. ** *p*< .01; *** *p*< .001.

Results supported Hypothesis 1 and are presented in Fig 1. The path between collective narcissism and belief in the hedonistic function of revenge was positive and significant, as was the path between belief in the hedonistic function of revenge and intergroup aggression. The indirect effect of collective narcissism on intergroup aggression was positive and significant *β* = .24, *z* = 5.06, *p* < .001, 95% CI [.17, .33]. The path between in-group satisfaction and belief in the hedonistic function of revenge was negative and significant, as was the indirect effect of in-group satisfaction on intergroup aggression via belief in the hedonistic function of revenge *β* = -.15, *z* = -3.23, *p* < .001, 95% CI [-.23, -.08]. The whole model was significant, *R*^2^ = .13, *z* = 3.68, *p* < .001.

Results also provided support for Hypothesis 2: Collective narcissism and in-group satisfaction acted as mutual suppressors of their opposite relations with belief in the hedonistic function of revenge. Suppression effects are summarized in Table 2. In Study 2 we sought to replicate these findings by using a more reliable measure of intergroup aggression: the voodoo doll task ([47]) and different target of intergroup aggression: Syrian refugees.

**Table 2:** Summary of suppression effects for Studies 1 and 2.

**Study 1**						
**IS as suppressor**	**Estimate**	**SE**	**Es. / SE**	**p-value**	**95% CI**	
**direct**	.29	.06	4.62	< .001	.17	.42
**indirect**	-.26	.05	-5.35	< .001	-.35	-.16
**total**	.04	.06	0.65	.517	-.07	.15
**CN suppressor**	**Estimate**	**SE**	**Es. / SE**	**p-value**	**95% CI**	
**direct**	-.40	.07	-6.02	< .001	-.53	-.27
**indirect**	.19	.04	4.38	< .001	.10	.27
**total**	-.21	.06	-3.7	< .001	-.32	-.10
**Study 2**						
**IS as suppressor**	**Estimate**	**SE**	**Es. / SE**	**p-value**	**95% CI**	
**direct**	.23	.07	3.13	.002	.11	.34
**indirect**	-.11	.04	-2.82	< .001	-.18	-.05
**total**	.11	.06	1.77	.076	.01	.22
**CN suppressor**	**Estimate**	**SE**	**Es. / SE**	**p-value**	**95% CI**	
**direct**	-.20	.07	-2.97	.003	-. 31	-.09
**indirect**	.12	.04	2.93	.003	. 06	.19
**total**	-.08	.06	-1.40	.161	-. 17	.02

IS = in-group satisfaction, CN = collective narcissism.

## Study 2

### Participants

Sample size for Study 2 was determined the same way as in Study 1 [44]. Regression coefficients were set to .20 for the path between the predictor and the mediator, and .15 for the path between the mediator and the outcome. Standard deviations of the predictors and mediators were set to .50. The smallest estimated sample size to discover the indirect effect was *N* = 246 (power = .80, alpha = .05). We oversampled to account for the missing data.

274 Polish university students participated in the study (232 female, 38 male, 4 gender missing data; Age: *M* = 25.70, *SD* = 8.11, range: 18–58) in exchange for research credits.

### Method

#### Materials

The response scale to all measures ranged from 1 (*totally disagree*) to 7 (*totally agree*). Both scales and items were presented in random order. **Collective narcissism** ([45]; α = . 82, *M* = 3.27, *SD* = 1.08), **in-group satisfaction** ([3, 48]; α = .90, *M* = 5.09, *SD* = 1.13) and **belief in the hedonistic function of revenge** (α = .95, *M* = 2.06, *SD* = 1.08) were measured the same way as in Study 1.

**Intergroup aggression** was measured with the voodoo doll task (VDT, [49]). Participants were presented with an outline of a human figure representing a Syrian refugee and asked to stab the doll with pins. Then, they indicated the number of pins they would stab into the doll using a slider depicting pins 0 to 51 (*M* = 1.46, *SD* = 5.81).

VDT is a measure of symbolic aggression that replicates the tendency humans have to attribute magical properties to objects. Participants are asked to ascribe characteristics of real individuals to an inanimate doll. VDT parallels other measures of aggression, has proved reliable over time, and displays appropriate sensitivity in laboratory settings [49]. The responses to the VDT do not signify ‘real’ aggression, because the victim does not experience direct harm. However, cognitive, emotional, and behavioural overlap exists between actual and symbolic forms of aggression and this task brought about results similar to actual aggression [14].

#### Results

Zero-order correlations between variables are summarized in Table 1. Overall, 78,3% of participants did not insert any pins, 18,4% inserted 1–10 pins, and 3,3% inserted more than 10 pins. The index of dispersion (also known as the variance-to-mean ratio) of the counts was 23.12 indicating overdispersion (i.e., variance of responses is greater than their mean), suggesting a negative binomial distribution of the counts [47].

**Fig 2:**
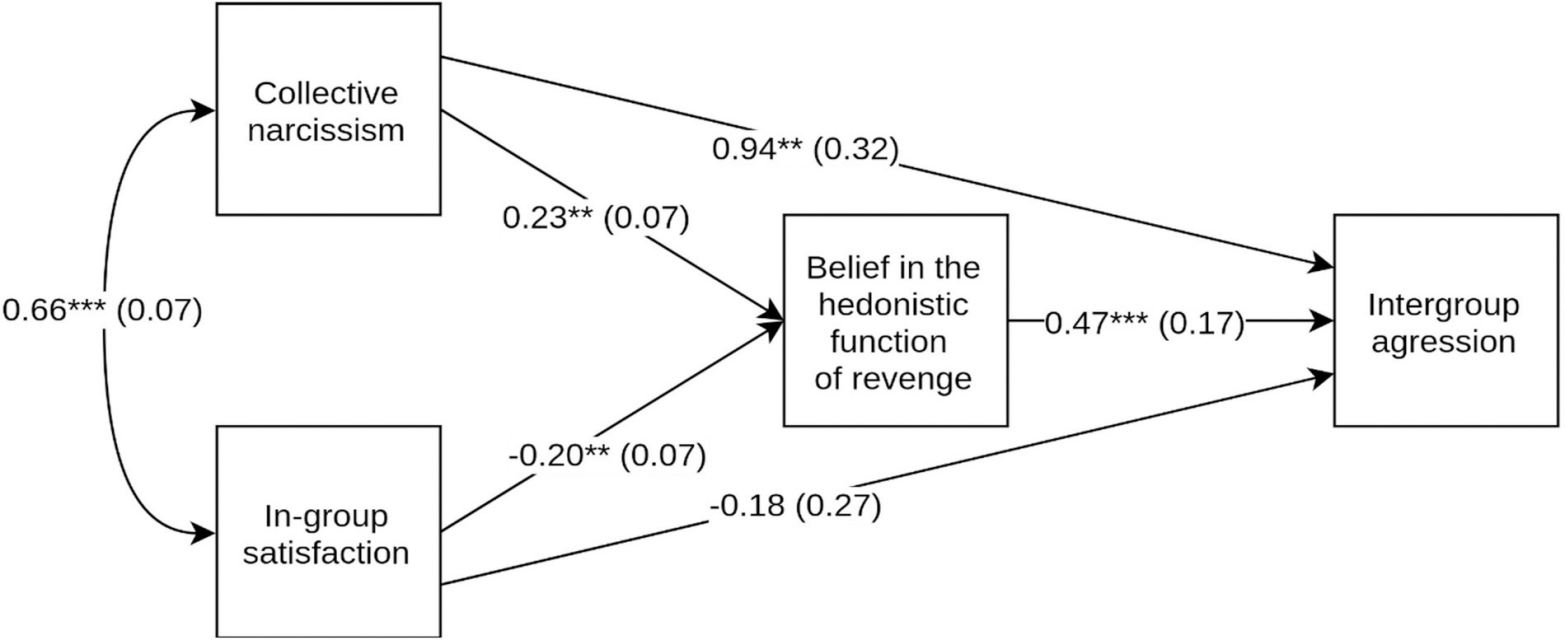
Unstandardized path coefficients, Study 2. ** *p*< .01; *** *p*< .001.

As in the Study 1, we analysed the hypothesized relation as a mediation model with two concurrent predictors in order to partial out variance shared by collective narcissism and in-group satisfaction. We used MPlus 8.4 with maximum likelihood estimation and a negative binomial distribution for the outcome. Results provided support for Hypothesis 1 and are presented in Fig 2. The indirect effect of collective narcissism on intergroup aggression via belief in the hedonistic function of revenge was positive and significant *β* = .11, *z* = 2.08, *p* = .038, 95% CI [.03, .20]. The indirect effect of in-group satisfaction on intergroup aggression via belief in the hedonistic function of revenge was negative *β* = -.09, *z* = -2.12, *p* = .038, 95% CI [-.18, -.03].

Results also supported Hypothesis 2: Collective narcissism and in-group satisfaction mutually suppressed their opposite associations with belief in the hedonistic function of revenge. Suppression effects are summarized in Table 2.

## Discussion

We examined whether two alternative beliefs about the in-group: Collective narcissism and in-group satisfaction predict aggression against out-group members via belief in the hedonistic function of revenge. Results from two studies found that collective narcissism was uniquely positively, whereas in-group satisfaction was uniquely negatively, associated with belief in the hedonistic function of revenge and, therefore, with intergroup aggression. These unique associations could only be observed when the covariation between collective narcissism and in-group satisfaction was partialled out. Thus, evidence is supportive for Hypothesis 1.

These results corroborate the results of previous studies untangling the opposite associations of collective narcissism and in-group satisfaction with attitudes towards out-group members: Collective narcissism is related to out-group derogation, whereas in-group satisfaction is linked to out-group tolerance [9–12, 50]. They also corroborate the recent findings on different emotional profiles associated with collective narcissism and in-group satisfaction [7]. Crucially, though, they provide further evidence that intergroup aggression may be an aspect of a general predisposition towards negative emotionality associated with collective narcissism.

In contrast, in-group satisfaction was negatively related to belief in the hedonistic function of revenge and intergroup aggression. Satisfied in-group members did not engage in aggressive actions against out-group members, believing that retaliatory aggression might be pleasant. These results align with the previous findings showing that in-group satisfaction is uniquely related to positive emotionality and well-being [7, 51, 52], and negatively associated with intergroup aggression [10]. They also corroborate previous research suggesting that in-group satisfaction in its residual form is uniquely associated with emotional resilience [11].

Our results also supported Hypothesis 2, that collective narcissism and in-group satisfaction suppress each other’s opposite associations with belief in the hedonistic function of revenge and intergroup aggression. Indeed, previous research has shown that collective narcissism and in-group satisfaction act as mutual suppressors of each other’s opposite associations with attitudes towards out-group members [9] and self-evaluations [7]. For instance, in-group satisfaction suppresses the negative relationship between low self-esteem and collective narcissism, whereas collective narcissism suppresses the positive relationship between high self-esteem and in-group satisfaction [10]. Similar effects have been found for sense of control [13], hostile attribution bias [9], and readiness to accept information about the past transgressions of the in-group [20].

Contrary to our expectations, reminding about past intergroup resentment did not interact with collective narcissism in predicting belief in the hedonistic function of revenge. One possible explanation is that the renouncement of the Polish-British alliance pact in 1939 is no longer perceived as resentment. Another possible explanation is that collective narcissists believe that revenge gives good feelings despite situational factors: They aggress against out-groups for often unrelated offenses [35] driven by the belief that revenge will make them feel good.

Overall, the present research suggests that intergroup aggression, similarly as interpersonal one, may be driven by desire to feel good [14]. More research is needed though to understand whether aggression against disliked or threatening outgroups is pleasant. If that is the case, then the pleasure of intergroup aggression may perhaps explain the perseverance of intergroup conflicts [48].

### Limitations and future directions

Our findings, although supportive for our Hypotheses, should be evaluated in light of several limitations. All the studies are correlational; therefore, we cannot make firm conclusions about causality of the observed effects. However, we provided a rationale for why beliefs about positive value of the in-group should determine (or not) belief in the hedonistic function of revenge and intergroup aggression. We also tested our hypothesized model against the model assuming that intergroup aggression mediated the relationship between belief in the hedonistic function of revenge and collective narcissism and in-group satisfaction (see S2 Table).

Results of Study 1, where intergroup aggression was measured as motivation to act aggressively against British [49], revealed higher regression coefficient when intergroup aggression mediated the relationship between both collective narcissism and in-group satisfaction and belief in the hedonistic function of revenge (see S2 Table). In Study 2 involving the voodoo doll task as a more direct measure of intergroup aggression [47], indirect effects had the same direction as in original analyses, but their path coefficients were smaller. We think this is because engaging in aggressive actions may increase belief in the rewarding function of aggression by either social learning [53] or by acquiring certain scripts that guide future aggressive behaviour [54].

Future studies would definitely benefit testing our hypotheses in experimental setups e.g. creating an intergroup situation that may increase desire for intergroup revenge or manipulating accessibility of the belief in the hedonistic function of revenge. Additional research is also needed to learn whether belief in the hedonistic function of revenge is indeed associated with pleasure after revengeful actions. If so, that would explain the perseverance of collective narcissistic aggressive actions and provide further evidence on whether revenge is factually a pleasant experience.

## Supporting information

S1 Table Effects coefficients for Study 1 with moderating effects of experimental condition on all paths. (TIF)

S2 Table Indirect effects coefficients for Studies 1 and 2 with intergroup aggression as mediator and belief in the hedonistic function of revenge as outcome. (TIF)
