# Correction: Opposite associations of collective narcissism and in-group satisfaction with intergroup aggression via belief in the hedonistic function of revenge

**DOI:** 10.1371/journal.pone.0250537

**Published:** 2021-04-15

**Authors:** The PLOS ONE Staff

The following information is missing from the Funding statement: The work on this article and open access publishing fee were financed by the Faculty of Psychology at the SWPS University of Social Sciences and Humanities in Warsaw from the departmental research fund awarded to BM. The publisher apologizes for the error.

Additionally, the ORCID iD is missing for the second author. Author Blazej Mrozinski’s ORCID iD is: 0000-0002-5423-2291 (https://orcid.org/0000-0002-5423-2291).
