# Supplementary figures and images for: Opposite associations of collective narcissism and in-group satisfaction with intergroup aggression via belief in the hedonistic function of revenge
Source: PLoS One. 2021 Mar 10;16(3):e0247814. doi: 10.1371/journal.pone.0247814 (PMC7946200; doi:10.1371/journal.pone.0247814)

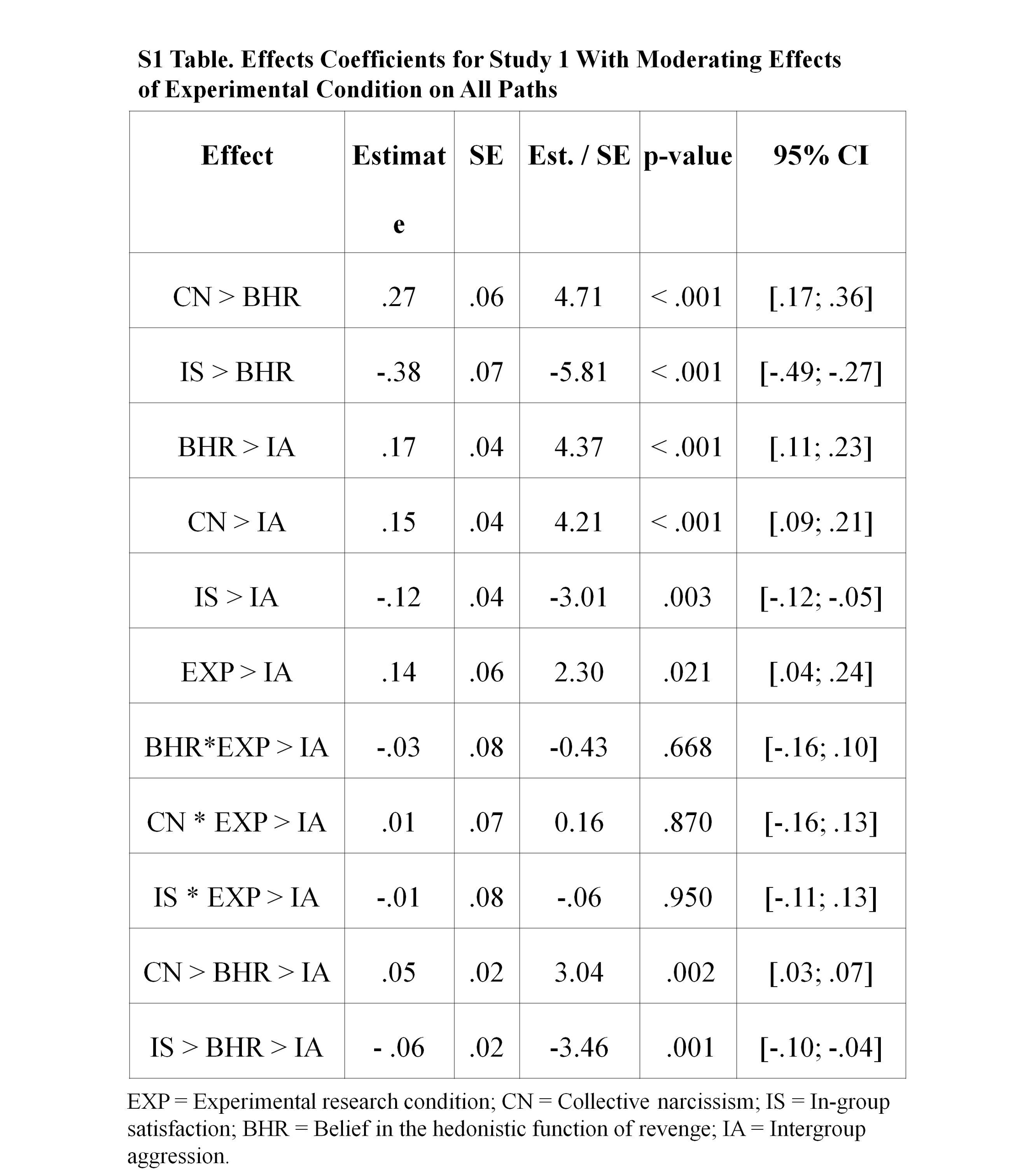

Supplement: S1 Table — (TIF) [file pone.0247814.s001.tif]

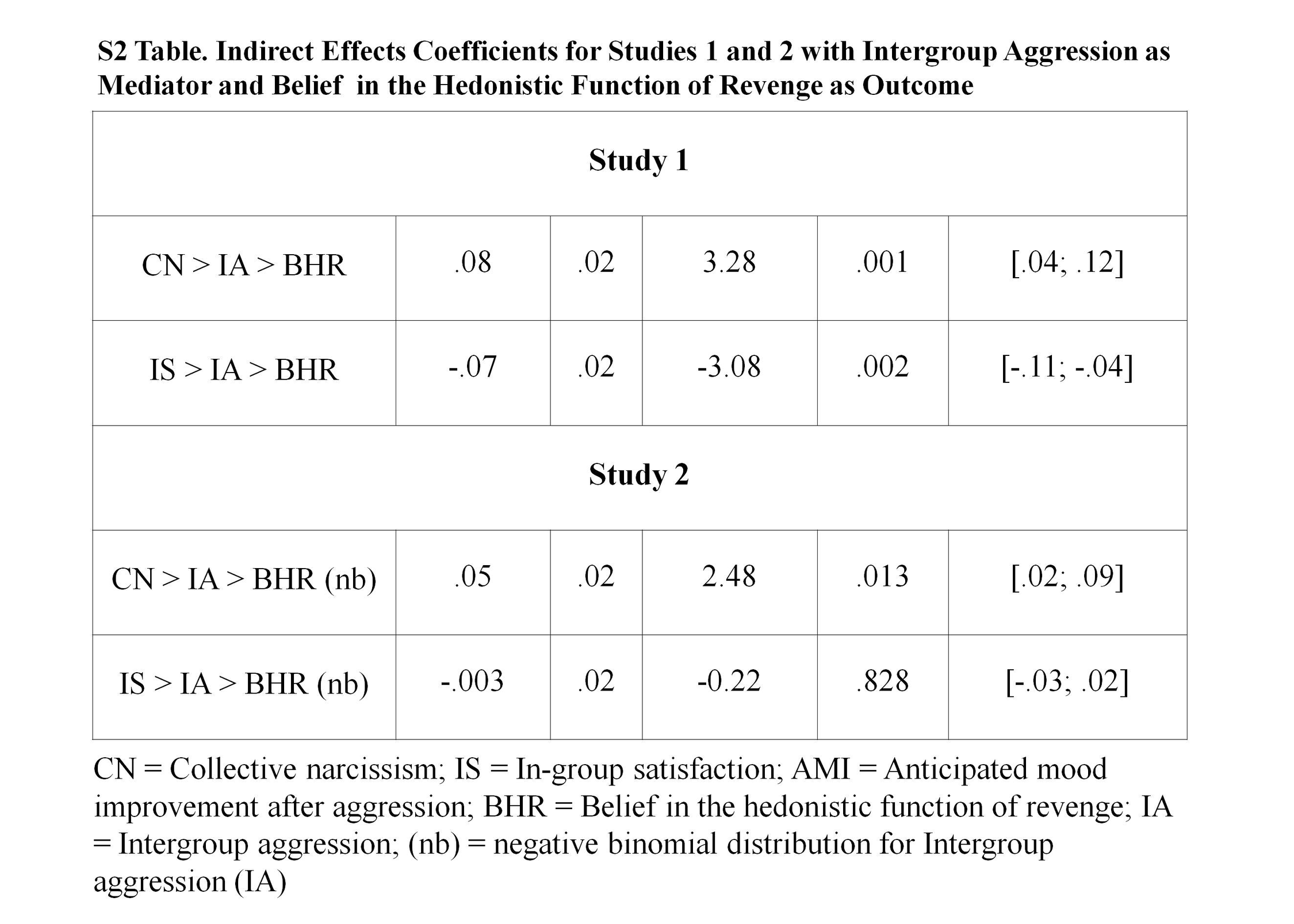

Supplement: S2 Table — (TIF) [file pone.0247814.s002.tif]
